# Preclinical and Clinical Evidence of Lurbinectedin in Ovarian Cancer: Current Status and Future Perspectives

**DOI:** 10.3389/fonc.2022.831612

**Published:** 2022-02-23

**Authors:** Lucia Musacchio, Carlo Maria Cicala, Vanda Salutari, Floriana Camarda, Maria Vittoria Carbone, Viola Ghizzoni, Elena Giudice, Camilla Nero, Maria Teresa Perri, Caterina Ricci, Francesca Tronconi, Giovanni Scambia, Domenica Lorusso

**Affiliations:** ^1^Department of Women and Child Health, Division of Gynecologic Oncology, Fondazione Policlinico Universitario A. Gemelli IRCCS, Rome, Italy; ^2^Comprehensive Cancer Center, Agostino Gemelli University Polyclinic (IRCCS), Rome, Italy; ^3^Marche Polytechnic University, Ancona, Italy; ^4^Department of Life Science and Public Health, Catholic University of the Sacred Heart, Rome, Italy

**Keywords:** ovarian cancer, lurbinectedin, platinum-resistant ovarian cancer, marine-derived drugs, DNA minor groove

## Abstract

Lurbinectedin is an antitumor agent belonging to the natural marine-based tetrahydroisoquinoline family which has shown very promising clinical activity with a favorable safety profile in many types of cancer. Preclinical evidence showed that lurbinectedin inhibits active transcription and binds to GC-rich sequences, leading to irreversible degradation of RNA polymerase II and generation of single- and double-strand DNA breaks and, as a consequence, apoptosis of tumor cells. In addition, lurbinectedin has demonstrated modulation of the tumor microenvironment and activity against cancer cells harboring homologous recombination DNA repair deficiency. Although considerable improvements have been made in the treatment of epithelial ovarian cancer, most patients with advanced disease experience recurrence with a dismal prognosis due to chemotherapy (mainly platinum) resistance. Platinum-resistant/refractory ovarian cancer remains a difficult-to-treat setting of disease, and currently, the exploration of new therapeutic approaches represents a main field of interest. Although the CORAIL phase III study did not meet its primary endpoint, the results suggest that lurbinectedin might be a valid alternative for patients that have exhausted therapeutic options. This article will focus on the clinical evidence, the most recent investigations, and the future perspective regarding the use of lurbinectedin in ovarian cancer.

## Introduction

Ovarian cancer (OC) is the eighth most common malignancy in women, with an estimated 313,959 new cases and 207,252 new deaths in 2020 (1). Due to a lack of early-stage detection, about 70% of patients present with advanced disease (FIGO stage III–IV) at diagnosis, and the mainstay of treatment is represented by radical surgery and platinum/paclitaxel chemotherapy. In the last few years, the introduction of anti-angiogenic agents and poly(ADP-ribose) polymerase inhibitors (PARPi) reshaped completely the outcomes of these patients, with remarkable improvements in terms of progression-free survival (PFS) (2–8) and overall survival (OS) (5). Despite the initial effectiveness of this approach, unfortunately, a large proportion of patients will experience disease relapse or progression. Treatment of recurrent OC for several years has been selected based only on the progression-free interval (PFI) after platinum-based chemotherapy. Recently, other considerations, i.e., the toxicity profile of the drugs, the genomic characteristics of the disease, the number of previous treatment lines, etc., play a role, in combination with PFI, in defining the most appropriate treatment at recurrence (9). Former platinum-resistant/refractory patients (patients who develop progressive disease during or within 6 months from platinum treatment completion) are usually managed with single-agent, non-platinum, chemotherapy between weekly paclitaxel, pegylated liposomal doxorubicin (PLD), gemcitabine, or topotecan with response rates (RR) around 10% and median OS of about 12 months (9). Therefore, the development of new agents in this setting represents a challenging field of interest. Lurbinectedin, a synthetic alkaloid originally derived from the marine tunicate *Ectenaiscidia turbinata*, has shown promising activity against platinum-resistant OC in preclinical models; in addition, some studies suggest that it possesses the capability to modulate the tumor microenvironment and to evoke anticancer immunity. Based on these lines of evidence and after the completion of a phase I trial assessing its tolerability and efficacy in advanced solid tumors (10), lurbinectedin was evaluated in platinum-resistant and platinum-refractory OC in a two-stage controlled phase II study, where a remarkable antitumor activity with a 23% overall response rate (ORR) (95% CI, 13%–37%) was reported (11). Based on these results, a randomized, controlled phase III trial of lurbinectedin or standard chemotherapy (PLD or topotecan) in platinum-resistant OC was designed. However, the primary endpoint of the CORAIL trial was not met, with no difference in PFS between the lurbinectedin arm and the standard chemotherapy arm (3.5 months, 95% CI 2.1–3.7 months vs. 3.6 months, 95% CI 2.7–3.8 months for lurbinectedin and standard chemotherapy, respectively). Moreover, compared with PLD or topotecan, lurbinectedin did not show a significant prolongation in OS nor a significant increase in ORR with a manageable toxicity profile (12). Although negative, the results of this trial suggest that lurbinectedin may be a reasonable therapeutic alternative in the management of platinum-resistant OC when other treatment options have been exploited. To explore its potential synergism and to enhance its therapeutic activity, lurbinectedin is currently being evaluated in several trials partnering with other agents such as PARPi (13).

## Background and Preclinical investigation

Lurbinectedin is a novel synthetic alkaloid structurally and functionally related to trabectedin, a marine-derived product of the ecteinascidin family, which is currently approved for the treatment of relapsed platinum-sensitive epithelial OC in combination with PLD (14). These two compounds share an analogous mechanism of action, which consists in the formation of a covalent bond with central guanines in specific nucleoside triplets located in the minor groove of the DNA molecules. These interactions lead to the formation of lurbinectedin–DNA adducts that eventually induce double-strand breaks (DSBs) in cancer cells and perturbations in the cell cycle; in *in-vitro* models, the exposure to lurbinectedin determines increased apoptotic rates in cancer cells, which occurs with a caspase-dependent pathway. As a result of these processes, lurbinectedin exerts strong *in-vitro* cytotoxic activities against multiple cancer cell lines, including OC, which has been confirmed *in vivo* through xenograft models of different human cancers (lung, ovary, colon, and stomach) (15).

It has been demonstrated that the cytotoxic activity of the members of the ecteinascidin family is dependent upon the mechanism of DNA nucleotide excision repair (NER). Selected cancer cell lines which are resistant to trabectedin show deficient xeroderma pigmentosum (XPG/ERCC4) gene expression, which is implicated in the NER pathway. Takebayashi et al. have shown that sensitivity to trabectedin can be restored by complementation with wild-type XPG, thus suggesting that trabectedin-induced cytotoxicity requires an intact NER mechanism. These authors propose that the adducts generated by the binding of trabectedin to the minor groove of DNA are recognized by the NER system, which ultimately leads to the formation of irreversible single-strand breaks and, consequently, to cell death (16). Interestingly, they observed that cisplatin-resistant cell lines display enhanced NER activity, which makes them more sensitive to trabectedin cytotoxicity.

The relationship between the NER system, platinum resistance, and the activity of trabectedin and lurbinectedin has been investigated in a preclinical model. After exposure to UV irradiation, which generates adducts in DNA molecules, platinum-resistant cell lines showed an increase in NER activity; nevertheless, when the same cell lines were treated with trabectedin or lurbinectedin, no cross-resistance to these agents was detected. Furthermore, a synergistic activity of the combination of lurbinectedin and cisplatin in cisplatin-resistant cell lines was observed (17). These findings have provided the bases to investigate the role of lurbinectedin in the setting of platinum-resistant OC.

The potential activity of lurbinectedin in platinum-resistant OC has been assessed in a preclinical model using a perpetuable orthotopic graft of a patient-derived epithelial ovarian cancer. In this murine model study (18), a cisplatin-resistant tumor graft was generated by serial cisplatin treatments and subsequent implantation of the post-treatment-derived tumor mass in mice. After the implant, mice were randomized to receive placebo, cisplatin, lurbinectedin, or the combination of the two agents. The results of this study showed that lurbinectedin is more effective than cisplatin in platinum-resistant OC and that the combination of cisplatin and lurbinectedin was more active than either single therapy in the context of platinum-resistant OC, again suggesting a synergistic activity of these two drugs. Of note, in cisplatin-sensitive OC, no significant benefit of the combined treatment was observed. The antitumor activity of lurbinectedin in OC was further corroborated by the analysis of the histological changes in tumor population: the highest grade of histopathological tumor regression was observed in the combination arm in both cisplatin-sensitive and cisplatin-resistant tumors.

Notably, lurbinectedin showed significant antitumor activity also against clear cell carcinoma (CCC) of the ovary, a relatively platinum-resistant OC subtype (19). In the study by Takahashi et al. (20), lurbinectedin inhibited tumor growth of platinum-resistant CCC cells both *in vitro* and *in vivo*; furthermore, when tested in combination with other antineoplastic agents, lurbinectedin showed synergistic activity with irinotecan, while its antitumor effects were enhanced when given in combination with the mammalian target of rapamycin-1 (mTORC1) inhibitor everolimus, which may represent a druggable target in CCC (21).

Along with its antitumor activity, lurbinectedin has also tumor microenvironment modulation properties which have been assessed in preclinical studies. When tested against human monocytes from healthy donors, lurbinectedin induces monocyte apoptosis *in vitro* and hampers proinflammatory activity by inhibiting the production of inflammatory chemokines such as CCL2 and CXCL8, which translates into a diminished monocyte migration. Furthermore, lurbinectedin exerts an anti-angiogenetic effect by inhibiting the generation of the vascular endothelial growth factor (VEGF). These effects have also been observed in cell lines and in *in-vivo* models, where treatment with lurbinectedin markedly reduced the amount of tumor-associated macrophages (TAM) and tumor vascularization (22). Tumor-associated inflammation is a well-recognized hallmark of cancer which contributes to tumor growth and survival; therefore, the activity of lurbinectedin in contrasting the tumor-associated proinflammatory cells makes this molecule of particular interest.

In addition, it has been recently shown that lurbinectedin may evoke anticancer immunity by inducing immunogenic cell death (ICD). In a preclinical study by Xie et al. conducted on osteosarcoma cell lines, treatment with lurbinectedin was associated with the stimulation of ICD as demonstrated by multiple cell modifications, such as the translocation of calreticuline (CALR) at the cell surface, the generation of an autocrine and paracrine response mediated by type I interferons, and the release of nuclear high mobility group box 1 (HMGB1), which is involved in tumor antigen recognition. Given these immunomodulatory effects, it has subsequently been investigated whether lurbinectedin may act in a synergistic fashion with immunotherapy in xenograft models, by sensitizing cancer cells to immune checkpoint inhibitors (ICIs). Treatment with a combination of both an anti-PD-1 and an anti-CTLA-4 antibody after exposure to lurbinectedin significantly extended the survival in murine models when compared with single ICI therapies; moreover, tumor-free mice that were rechallenged with the same cancer type show tumor rejection, indicating that the combination of lurbinectedin and immunotherapy may generate immunological memory (23).

## Published Clinical Data

A recent phase I trial investigated the recommended phase II dose (RP2D) of cisplatin administered in combination with lurbinectedin, with or without aprepitant (group A and group B, respectively) in patients with advanced solid tumors, including OC. The secondary objectives of the study were the characterization of safety profile, pharmacokinetics, and preliminary antitumor activity. All patients were treated with 60 mg/m^2^ cisplatin intravenous (i.v.) infusion followed by lurbinectedin i.v. infusion at escalating doses on day 1 every 3 weeks (q3wk). For patients in group A, the recommended dose was cisplatin 60 mg/m^2^ plus lurbinectedin 1.1 mg/m^2^, while for group B, the recommended dose was cisplatin 60 mg/m^2^ plus lurbinectedin 1.4 mg/m^2^. The most frequent grade ≥3 adverse events were hematological [neutropenia (41%), lymphopenia (35%), leukopenia (24%), thrombocytopenia (18%)] and fatigue (35%) in group A (n = 17) and neutropenia (50%), leukopenia (42%), lymphopenia (29%), and fatigue (13%) and nausea (8%) in group B (*n* = 24). Four patients (2 in each group) had a partial response and 14 patients (4 in group A and 10 in group B) achieved a stable response. No signs of activity were reported in the cohort of OC patients as well as in the group of patients receiving aprepitant, and the combination of lurbinectedin and cisplatin was considered highly toxic (24). Another multicenter, open-label, phase I study evaluated the recommended dose (RD) of the combination of lurbinectedin and gemcitabine in patients with advanced solid tumors. Forty-five patients were treated between May 2011 and May 2013 and received lurbinectedin 3.5 mg flat dose (FD)/gemcitabine 1,000 mg/m^2^. Dose-limiting toxicities (DLTs) were mostly hematological and resulted in the expansion of a lower dose level (lurbinectedin 3.5 mg FD/gemcitabine 800 mg/m^2^); 19 patients at this dose level were evaluable but >30% reported DLT and >20% had febrile neutropenia. On the contrary, DLT was observed in 11 patients treated with lurbinectedin 3.0 mg FD/gemcitabine 800 mg/m^2^, which was defined as the RD. Nine of 38 patients were evaluable for response according to RECIST 1.1, with 3% complete responses and 21% partial responses, with an ORR of 24% (95% CI, 12%–40%). Eleven patients (29%) had disease stabilization for at least or more than 4 months. The median duration of response was 8.5 months and the median PFS was 4.2 months (95% CI, 2.7–6.5 months). This schedule is generally well tolerated and has reported antitumor activity in several advanced solid tumors (25). Recently, the results of a phase I study, designed to evaluate the safety and toxicity of lurbinectedin in combination with olaparib in patients with advanced solid tumors without standard therapeutic alternatives, were published. In total, 20 patients with OC, endometrial cancer, and uterine leiomyosarcoma were enrolled in this 3 + 3 dose-escalation study. The RP2D was lurbinectedin 1.5 mg/m^2^ on day 1 and olaparib capsules 250 mg BID on days 1–5 of a 21-day cycle. The study did not report complete or partial responses, but disease control rate was achieved in 60% of patients. The most common, mainly grade 1–2, adverse events were asthenia (55%), nausea (55%), vomiting (50%), constipation (45%), abdominal pain (40%), neutropenia (35%), and anemia (35%) (13). The safety and the efficacy of single-agent lurbinectedin were also evaluated in a two-stage, controlled, randomized, multicenter phase II study trial. The primary endpoint was ORR by the RECIST and/or GCIG criteria. The exploratory first stage (*n* = 22 patients) confirmed the activity of lurbinectedin as a single agent at 7.0 mg flat dose q3wk. The second stage (*n* = 59) was randomized and controlled versus topotecan on days 1–5 q3wk (1.50–0.75 mg/m^2^) or weekly (4.0–2.4 mg/m^2^). The ORR was 23% (95% CI, 13%–37%) for lurbinectedin with a median duration of response of 4.6 months (95% CI, 2.5–6.9 months), with 23% (95% CI, 0%–51%) of the responses lasting 6 months or more. Ten of the 12 confirmed responses were reported in the 33 platinum-resistant patients [ORR = 30% (95% CI, 16%–49%)]. No responses were reported among the 29 patients treated with topotecan. The median PFS was 4.0 months (95% CI, 2.7–5.6 months) for all lurbinectedin-treated patients and 5.0 months (95% CI, 2.7–6.9 months) for patients with platinum-resistant disease. Specifically, in the second randomized stage, the median PFS was significantly longer with lurbinectedin 3.9 months (95% CI, 2.5–5.7 months) versus 2.0 months (95% CI, 1.4–2.8 months) with topotecan (*P* = 0.0067). The median OS was 9.7 months (95% CI, 7.7–19.3 months) with lurbinectedin and 8.5 months (95% CI, 3.3–15.6 months) with topotecan (*P* = 0.2871).

Myelosuppression was the most frequent adverse event (AE). In the lurbinectedin arm, grade 3/4 neutropenia and thrombocytopenia were observed in 85% and 33% of patients, respectively, while in the topotecan arm, grade 3/4 neutropenia occurred in 38% of patients and grade 3/4 thrombocytopenia in 24% of patients (11).

Recently, the results of a phase III, multicenter, randomized trial, evaluating the efficacy of lurbinectedin with respect to PLD or topotecan in platinum-resistant ovarian cancer patients, were published. In this trial, patients were randomized in a 1:1 ratio to receive lurbinectedin 3.2 mg/m^2^ i.v. infusion q3wk in the experimental arm or PLD 50 mg/m^2^ i.v. infusion q4wk or topotecan 1.50 mg/m^2^ i.v. infusion days 1–5 q3wk in the control arm. Performance status (PS) (0 vs. ≥1), prior PFI (1–3 vs. >3 months), and prior chemotherapy lines (1–2 vs. 3) were the stratification factors. The primary endpoint was PFS evaluated by an independent review committee according to RECIST 1.1. Two hundred and twenty-one women were randomized in the lurbinectedin arm and 221 patients in the control arm (127 of them received PLD and 94 patients were treated with topotecan). With a median follow-up of 25.6 months, the median PFS was 3.5 months (95% CI, 2.1–3.7) in the lurbinectedin arm and 3.6 months (95% CI, 2.7–3.8) in the control arm (stratified log-rank *P* = 0.6294; HR = 1.057), respectively. The safety of lurbinectedin was considered manageable: grade ≥3 treatment-related AEs were the most frequent in the control arm: 64.8% vs. 47.9% (*P* = 0.0005), mainly due to hematological toxicities. The most common non-hematological grade ≥3 AEs were fatigue (7.3% of patients) and nausea (5.9%) in the experimental arm, while mucosal inflammation (8.5%) and fatigue (8.0%) were the most common non-hematological grade ≥3 toxicity in the control arm (12).

## Conclusion

Platinum-resistant and platinum-refractory OCs have a dismal prognosis and treatment options in these patients are limited (9). Lurbinectedin demonstrated antitumor activity in the phase II study by Poveda et al., with an ORR of 23% (95% CI, 13%–37%), a median PFS of 4.0 months (95% CI, 2.7–5.6 months), and a median OS of 10.6 months (95% CI, 9.5–19.1 months) (11). Unfortunately, the phase III, randomized, multicenter CORAIL study failed to demonstrate the superiority of this agent in terms of PFS when compared with topotecan and PLD in platinum-resistant OC patients (stratified long-rank *P* = 0.6294; HR = 1.057) (13). However, some consideration may help in the interpretation of the CORAIL trial results. In the CORAIL trial, the patients were older with respect to the previously reported phase II trial (patients ≥65 years, 43% vs. 27%), more heavily pretreated (three prior chemotherapy lines in 23% vs. 12%), had a shorter median PFI (3.9 vs. 4.6 months), and had reported fewer responses to the last platinum therapy (31% vs. 76%). Additionally, a larger proportion of patients presented ascites (27% vs. 18%), which seems to abolish the activity of lurbinectedin, inhibiting its cellular uptake (26). Moreover, the dosages of the two standard arm regimens were poorly used in clinical practice because of unmanageable toxicity, and in particular, the dosage and schedule of topotecan was different in the two trials, with more patients treated with the less effective weekly regimen in the phase II than in the phase III. Regardless of the results, the phase III trial reported an activity of lurbinectedin at least overlapping to that registered with the most used standard regimens, with a better toxicity profile. In this context, in our opinion, it remains unclear the real benefit of lurbinectedin for these patients and the place, if any, of lurbinectedin in the treatment armamentarium of platinum-resistant OC disease.

Moreover, lurbinectedin induces the generation of double-strand DNA breaks, with consequent cell apoptosis, and reduces tumor-associated macrophages and the inflammatory microenvironment through inhibition of inflammatory factors (27).

Since these DNA double-stand breaks are processed through homologous recombinant repair (HRR), lurbinectedin is associated with activity in HRR-deficient cells, and the molecular data in the CORAIL trial seem to suggest that patients with tumor harboring BRCA mutations had a longer survival compared with those without BRCA mutations when treated with lurbinectedin. Additionally, a recent multicenter phase II trial showed a notable response and survival advantage in BRCA 1/2 breast cancer patients treated with lurbinectedin (28). Based on these results, the combination of olaparib, an inhibitor of DNA damage repair (DDR), with a DNA-damaging agent such as lurbinectedin seems an interesting approach to maximize the effect of DNA damage, and in a phase I study, the combination of these agents showed antitumor activity with 60% of DCR in patients with solid tumors (13).

Preclinical evidence has suggested that the simultaneous inhibition of multiple DNA repair mechanisms, along with the DNA damage induced by ectenaiscidins, might enhance the activity of these drugs. Lima et al. have shown that the treatment with lurbinectedin and trabectedin activates both ATM and ATR pathways in OC cell lines (29). The combination of an ectenaiscidin with an inhibitor of ATM or ATR did not provide a significant increase of the cytotoxicity of lurbinectedin; however, the simultaneous inhibition of both ATM and ATR resulted in a marked increase of lurbinectedin-induced cell death (29). This seems to suggest that the two mechanisms may, at the same time, be overlapping and complementary and that the dual inhibition of these pathways may significantly enhance the activity of ectenaiscidins. In addition, Riabinska et al. have demonstrated that, in human and murine cancer cells treated with genotoxic chemotherapy, ATM depletion leads to strong addiction on DNA-PKcs.

In their paper, Riabinska et al. showed that the inhibition of DNA-PK in ATM-defective cells leads to apoptotic death (30). These lines of evidence may provide the basis to combine lurbinectedin with other inhibitors of the DNA repair machinery such as berzosertib, an ATR inhibitor, which is currently been tested in a phase I trial in advanced solid tumors.

In conclusion, lurbinectedin showed antitumor activity in platinum-resistant OC patients with a favorable safety profile, suggesting that this agent should continue to be considered as an option in this setting of disease. Moreover, recent studies have shown that combining lurbinectedin with DNA repair inhibitors, i.e., PARPi, seems particularly promising in HRR ovarian and breast cancer patients.

## Author Contributions

Conceptualization: DL. Data curation: LM and CC. Investigation: LM and CC. Methodology: DL and LM. Project administration: DL. Supervision: DL. Writing—original draft: LM, CC, and DL. All authors acquired the data, revised the manuscript, and approved the final version of the manuscript.

## Conflict of Interest

DL reports research funding from Clovis, GSK, and MSD; personal interests with AstraZeneca, Clovis Oncology, GSK, PharmaMar, and MSD; and financial interests with Clovis, Genmab, GSK, and MSD; and is part of the Board of Directors, GCIG (Gynecologic Cancer Inter Group). VS reports honoraria from GSK, PharmaMar, Roche, MSD, EISAI, Clovis, Oncology, and AstraZeneca. GS reports research support from MSD and honoraria from Clovis Oncology and is a consultant for Tesaro and Johnson & Johnson.

The remaining authors declare that the research was conducted in the absence of any commercial or financial relationships that could be construed as a potential conflict of interest.

## Publisher’s Note

All claims expressed in this article are solely those of the authors and do not necessarily represent those of their affiliated organizations, or those of the publisher, the editors and the reviewers. Any product that may be evaluated in this article, or claim that may be made by its manufacturer, is not guaranteed or endorsed by the publisher.
